# Directing coral larval settlement in coral aquaculture for reef restoration

**DOI:** 10.1038/s41598-026-37592-x

**Published:** 2026-02-05

**Authors:** Nico D. Briggs, Andrew P. Negri, Elsa Antunes, Matthew Drane, Andrea Severati, Florita Flores

**Affiliations:** 1 Australian Institute of Marine Science, PMB No. 3, Townsville MC, Townsville, QLD 4810 Australia; 2 Division of Research & Innovation, AIMS@JCU, James Cook University and Australian Institute of Marine Science, PMB No. 3, Townsville MC, Townsville, QLD 4810 Australia; 3 College of Science and Engineering, James Cook University, Townsville, QLD 4811 Australia

**Keywords:** Great Barrier Reef, Ceramic 3D printing, Microtopography, Chemical cues, Neuropeptides, Biological techniques, Biotechnology, Ecology, Ecology, Microbiology, Ocean sciences

## Abstract

Optimising coral early life stages, particularly larval settlement, remains a challenge for coral aquaculture. Settlement typically relies on biologically conditioned substrates, such as crustose coralline algae (CCA) or microbial films, which are difficult to scale for mass-production. To overcome this bottleneck, we evaluated chemical and physical alternatives that enable spatial control of directing larval settlement. Larval responses from 14 coral species were tested against known and potential chemical inducers, including neurotransmitters and neuropeptides. Next, we investigated the efficacy of a set of known chemical inducers and microtopography to direct larval settlement. The neuropeptide Hym-248 was the most effective, inducing settlement in seven acroporid species. Embedding soluble inducers (CCA extract and Hym-248) in agar hydrogels within ceramic cubes successfully induced and directed settlement of *Acropora kenti*. Similarly, CCA-conditioned ceramic protrusions, with and without microtopographic pores, achieved > 99% settlement on or adjacent to protrusions. These findings demonstrate that immobilised chemical cues can precisely localise larval settlement on unconditioned substrates and that conditioning only small, discrete 3-D printed features is as effective as conditioning entire tiles. This approach substantially reduces aquarium space and resource requirements, offering a scalable, cost-effective pathway to enhance the efficiency of sexually propagated coral aquaculture in reef restoration.

**Supplementary Information:**

The online version contains supplementary material available at 10.1038/s41598-026-37592-x.

## Introduction

Coral reefs are under increasing pressure from multiple local stressors, including poor water quality, coastal development and overfishing, as well as global climate change stressors, such as ocean warming and acidification, which compromise their ability to provide critical ecosystem services^1–3^. Between 2009 and 2018, global coral cover declined by 14%, primarily due to the increasing frequency and reoccurrence of large-scale bleaching events^4^. This global decline has driven the emergence of reef restoration efforts employing a range of intervention strategies. One such approach is coral aquaculture, or coral farming, which involves the rearing of corals, and has been practiced for several decades at different scales in countries, such as Australia, USA, Indonesia, Japan, and the Philippines^5,6^. Most coral aquaculture and restoration programs use asexually propagated fragments or microfragments, in which coral colonies are fragmented and manually outplanted onto degraded reefs to establish new colonies^7,8^ However, this method is labour intensive and requires a large supply of healthy donor colonies, limiting its sustainability and scalability. A more cost-effective and sustainable alternative involves the use of sexually propagated corals, as adult colonies can produce tens of thousands of coral larvae, supporting greater genetic diversity, and offering better prospects for upscaling coral aquaculture^9–12^.

Coral gametes develop into free-swimming larvae after fertilisation and possess mechanisms that guide them to undergo settlement, the transition from the water column to substrate attachment and metamorphosis into a flattened disk-like polyp^13^. These mechanisms include receptors that recognise biochemical cues which signal attachment and metamorphosis. Settlement cues often originate from microbial biofilms^14–16^ and/or crustose coralline algae (CCA)^17,18^. However, larval responses to these chemical cues are frequently species-specific, such that different CCA species show variable levels of success in inducing larval settlement^19–22^, and preferences for settlement substrates may also depend on environmental conditions influenced by light, water movement, or sediment load^23,24^. A common method to promote larval settlement involves providing a substrate that has developed a natural biofilm (through a process termed ‘conditioning’), often including CCA^16^. The optimal conditioning period depends on substrate material, tank community composition, and environmental parameters such as temperature, light, water flow, and the presence of biological inputs like grazing fish and invertebrates^25,26^.

A variety of conditioned settlement substrates have been applied in coral seeding, including terracotta, ceramic, polyvinyl chloride, concrete mixed with coral rubble, and concrete^5,27–30^—the latter now widely adopted as the standard material used in reef seeding devices by restoration groups, such as the Reef Restoration and Adaptation Program^31,32^. Concrete tiles (28 × 28 cm), composed of smaller detachable ‘tabs’ (20 × 20 tabs, 1.4 cm × 1.4 cm), are conditioned in aquaria for approximately six weeks to promote the development of biological settlement cues. After settlement, tabs with multiple coral spat (settled coral larvae) are then separated and inserted into larger ceramic seeding devices for outplanting^32^. However, this process incurs substantial costs in terms of time, labour, and facility requirements^33^, particularly the space needed for conditioning and storing of large quantities of tiles prior to settlement, and the management of competitive algal growth to ensure spat survival^25,34^. Biological conditioning can be upwards of 12 weeks depending on the maturity (i.e., covered in CCA) of the conditioning tank^33^. Bypassing the conditioning step can save time, labour, aquarium space and eventual costs required to condition the quantities of settlement substrates necessary for reef restoration efforts. Furthermore, biological conditioning of concrete substrates is often irregular, leading to settlement patterns that can be sporadic, highly localised, or along uneven gradients, thus making portions of the substrate unusable^33^. Directing coral larvae to a centrally located position on detachable concrete tabs would reduce larval loss during tab separation, and ensure more useable coral spat units, improving the likelihood of scalable recruit production methods.

There is also potential to reduce the costs and logistical challenges associated with traditional biological conditioning by using alternative methods to induce larval settlement. Dusting settlement substrates with crushed CCA is an easily prepared substitute for traditional CCA conditioning^11^, and soluble CCA extracts have long been used to induce high rates of settlement in some corals^17,35^. CCA extracts are typically prepared from ethanol- or methanol-based extractions, and exhibit potent morphogenic characteristics when introduced to coral larvae^18,35,36^. In addition to chemical extracts, bacterial isolates from live CCA have induced both complete and partial settlement in *Acropora, Pocillopora,* and *Porites* ^14,37,38^, and other bacteria in microbial biofilms commonly found on reef substrates are associated with coral settlement^15,39,40^. Non-acroporid species have been observed to preferentially settle on rubble conditioned with biofilms rather than on CCA ^21^. This preference may reflect a mismatch between the niche chemical cues utilised by these species and settlement signals associated with commonly collected CCA species, which are typically harvested from shallow depths for ease of access. Microbial biofilms are increasingly recognised as a source of morphogenic chemicals, with several active chemicals successfully isolated from bacterial strains. For example, tetrabromopyrrole (TBP) has been shown to induce larval metamorphosis in both Pacific and Caribbean coral species^41–43^. The pigment cycloprodigiosin (CYPRO) also triggers larval metamorphosis by providing a supply of hydrogen peroxide to coral larvae through a light-dependent reaction^44^.

Neurotransmitters^45^ and neuropeptides^46–49^ can also function as alternative metamorphosis inducers. Moeller et al.^45^ demonstrated that dopamine, glutamic acid and epinephrine induced settlement in the brooding coral *Leptastrea purpurea.* Peptides from the GLWamide family isolated from the sea anemone *Anthopleura elegantissima* and the hydroid *Hydra magnipapillata* were found to induce metamorphosis in larvae of the hydroid *Hydractinia serrata*^50^. These signalling pathways have been retained in some coral species, particularly within the genus *Acropora*^47^. Several studies have reported successful metamorphosis in *Acropora* spp. larvae and other anthozoans when exposed to low concentrations of GLWamide neuropeptides^21,46–48^. Chemical biocoatings, such as SNAP-X^51^, can also induce coral settlement by slowly releasing bioactive molecules into the surrounding microenvironment. However, the ecological relevance of all these chemicals in directly inducing larval settlement in situ is not confirmed, as some neuropeptides and synthesised chemical inducers trigger metamorphosis without attachment in many of the species investigated.

In addition to chemical cues, physical settlement features, such as microtopographies, also influence larval settlement behaviour^27,52–55^. Larvae often settle in crevices or interstitial spaces^13,56^, within topographical features that match their body size^57^, or on colour-specific substrates^58,59^. Although chemical, physical and microbial settlement cues have all been studied, outcomes are often inconsistent, vary across species and cue types, and rarely scale beyond a few cm^2^. While Jorissen et al.^20^ localised settlement on the surface of *Titanoderma prototypum* and general settlement responses to microtopographic features have been documented, methods to precisely direct coral larval settlement remain scarce.

Enhancing the efficacy and cost-effectiveness of sexually propagated coral deployment in reef restoration requires optimising both settlement success and spatial uniformity. In this study, we aimed to achieve this through three approaches. First, we investigated the efficacy of known and potential chemical inducers, including neuropeptides and neurotransmitters, to induce small-scale settlement across 14 coral species. Next, we embedded effective chemical cues into agar hydrogels, which were inserted into the wells of ceramic perforated cubes affixed to unconditioned concrete tiles to assess whether these cues could influence larval settlement behaviour. Finally, we evaluated whether localised biological conditioning and/or physical features could direct settlement. Achieving reliable induction and direction of larval settlement without the need for time- and resource-intensive substrate conditioning could significantly improve the scalability of coral conservation aquaculture.

## Methods

### Organism collection and coral larval cultures

Fecund colonies (up to 40 cm diameter) of 14 coral species (Supplementary Table 1) were collected off Esk Island (18°46′06″S, 146°31′19″E) and Falcon Island (18°76′71″S, 146°53′42″E) within the Palm Islands Group, and Davies Reef, Great Barrier Reef, Australia (18°83′26″S, 147°63′32″E) from 25 to 28 October and 22–27 November 2023 (Experiment 1). *Acropora kenti*^60^ colonies were collected on 10 November 2024 from John Brewer Reef (18°63′17″S, 147°02′73″E) (Experiments 2 and 3). Fragments of crustose coralline algae (CCA), *Porolithon* cf. *onkodes* were collected from Davies Reef (18°83′26″ S, 147°63′32″ E; Experiments 1–3). Corals and CCA were collected under Great Barrier Reef Marine Park Authority (GBRMPA) permits G23/49085.1, G21/45348.1, G23/49457.1, and G21/38062.1 (Supplementary Table 1).

Corals and CCA were transported to the National Sea Simulator (SeaSim) at the Australian Institute of Marine Science (AIMS) in Townsville, Australia. Corals were maintained in semi-recirculating outdoor aquaria (1000 L) under ambient light and temperature conditions matching those of the collection sites (max 700 µmol photons m^-2^ s^-1^ photosynthetically active radiation (PAR); ~ 28 °C). CCA were maintained in semi-recirculating indoor aquaria (270 L) at max 50 PAR and ~ 28 °C. Gametes were collected from parental colonies, fertilised, and cultured at < 1 larvae mL^-1^ in 70 L or 500 L fibreglass flow-through tanks (1.5 turnovers d^-1^; Supplementary Table 1) with 1 µm filtered seawater (FSW; 28 °C) as described in other experiments which successfully reared coral larvae at SeaSim^19,21^.

### Preparation of chemical cues

A range of chemicals known to induce larval metamorphosis and settlement were selected based on previous studies. These included ethanolic extracts of CCA^22,35,36^,crushed CCA^11^,GLWamide neuropeptides^21,46,47,61^,and neurotransmitters (dopamine and epinephrine;^45^.

#### CCA extract

The CCA *Porolithon* cf. *onkodes* (herafter, termed *Porolithon*) has been shown to induce settlement in larvae of the genus *Acropora*^19,22^. Additionally, this CCA is identifiable and can be readily harvested from the reef crest^33^. Ethanolic extractions of *Porolithon* cf. *onkodes* were prepared following methods described in^22,36^. Briefly, 150 g of material was obtained from the outer ~ 3 mm of the CCA thallus and ground with a mortar and pestle. The crushed material was mixed with 150 mL 100% ethanol (> 99% purity, Univar) and agitated horizontally on a roller for 2 h at room temperature (25 °C). The ethanol extract was decanted and the remaining CCA paste was re-extracted using the same method. Combined extracts were vacuum filtered (Whatman GF/F, 0.7 µm), then concentrated under vacuum to yield a final stock concentration equivalent to 0.5 g CCA mL^−1^ ethanol. Extracts were stored at − 20 °C.

#### Crushed CCA

Fragments from the outer ~ 3 mm of the thallus of *Porolithon* (10 × 10 mm) were ground into a slurry using a mortar and pestle, freeze dried for 24 h (Freezone, Labconco Corp., USA), and then ground to a fine powder via mortar and pestle. Crushed CCA was stored in − 20 °C until experimental use.

#### Neuropeptides and neurotransmitters

Structure, sequence and purity of neuropeptides are listed in Supplementary Table 2. Dopamine (≥ 98% purity) and epinephrine (≥ 98% purity) were sourced from Sigma-Aldrich (USA). All neurotransmitters and neuropeptides were dissolved in Milli-Q water to make stock concentrations of 5 mM, 10 mM, and 20 mM. The neuropeptide Hym-248, belonging to the GLWamide family, is a known chemical inducer of larval settlement^46–48^. The GLWamide family has six other members that share the same C-terminal structure as Hym-248, which were investigated for inducing larval settlement in this study (Supplementary Table 2,^62^). However, we were unable to obtain Hym-301 and therefore that peptide was not tested.

### Efficacy of chemical inducers across taxa (Experiment 1)

Larval settlement assays were performed with 14 species across three families (Supplementary Table 1) in sterile 6-well polystyrene cell culture plates (12 mL, Nunc) under 27–28°C and ~ 5–10 PAR, conditions similar to the stock larval cultures. Ten coral larvae of each species (4–7-day-old) were transferred using disposable transfer pipettes into separate wells with 10 mL FSW and the cue to be tested. Treatments included: filtered seawater (0.2 µm), referred to as FSW (negative control); live CCA *Porolithon* cf. fragment of approximately 25 mm^2^ (positive control)^19,35^, fragment of calcified reef rubble with live biofilm (~ 25 mm^2^)^21^,dopamine and epinephrine (1 µM and 10 µM)^45,63^, and six GLWamide neuropeptides (1 µM and 10 µM; Hym-38, Hym-53, Hym-54, Hym-248, Hym-249, and Hym-331)^21,46,47^. Each treatment was run in triplicate wells except for the negative control which was performed in six replicate wells.

Larvae were assessed for settlement competency (> 70% settlement) the day prior to experimentation using a fragment of CCA *Porolithon* and reef rubble^19,21^. There were six replicates for negative controls and three replicates for each inducer treatment. Each coral species was tested individually with each treatment. Metamorphosis was assessed using a dissecting microscope after approximately 24 h and considered successful when free-swimming larvae had metamorphosed into squat, firmly attached, disc-shaped structures with flattening of the oral-aboral axis^35^. Mean percent settlement was calculated for each inducer treatment by averaging the percentage of larvae that successfully settled across treatment replicates, and ± SE was calculated from these values.

### Embedded chemical inducers under flow-through (Experiment 2)

While larval settlement assays in well plates are useful for evaluating responses to specific chemical or biological cues, their limited water volume and restricted flow conditions can constrain larval behaviour, resulting in settlement patterns that do not accurately reflect those observed in larger-scale systems used for mass settlement on concrete surfaces in reef restoration programs. To evaluate the performance of chemical inducers in directing larval settlement at a larger scale, Experiment 2 was conducted in 15 L flow-through aquaria, with immobilised chemical inducers on precise locations on unconditioned substrates. Ethanolic extracts of CCA and the neuropeptide Hym-248 were selected as active chemical inducers based on results from Experiment 1 and their reported activity in previous studies^21,22,46^. Both Hym-248 and CCA extract successfully induced settlement but, being water soluble, would dissolve and dilute rapidly in flow-through systems. To mitigate this, we embedded the inducers in bacteriological agar (ChemSupply Australia) to retain and localise their activity. Solutions of Hym-248 and CCA extract were separately mixed into liquid agar heated to 85 °C to facilitate dispensing. Hym-248 was first dissolved in Milli-Q water to final concentrations of 5 mM or 10 mM, then combined with 3% agar (1:1 v/v) to yield a final agar concentration of 1.5%. CCA extract in ethanol was dried under nitrogen to remove residual solvent (as ethanol can be toxic to coral larvae)^64^ and then incorporated into heated 1.5% agar. Crushed CCA suspended in 1.5% agar was also tested as a potential alternative to CCA extract, requiring less preparation.

#### Perforated ceramic cubes

**Fig. 1:**
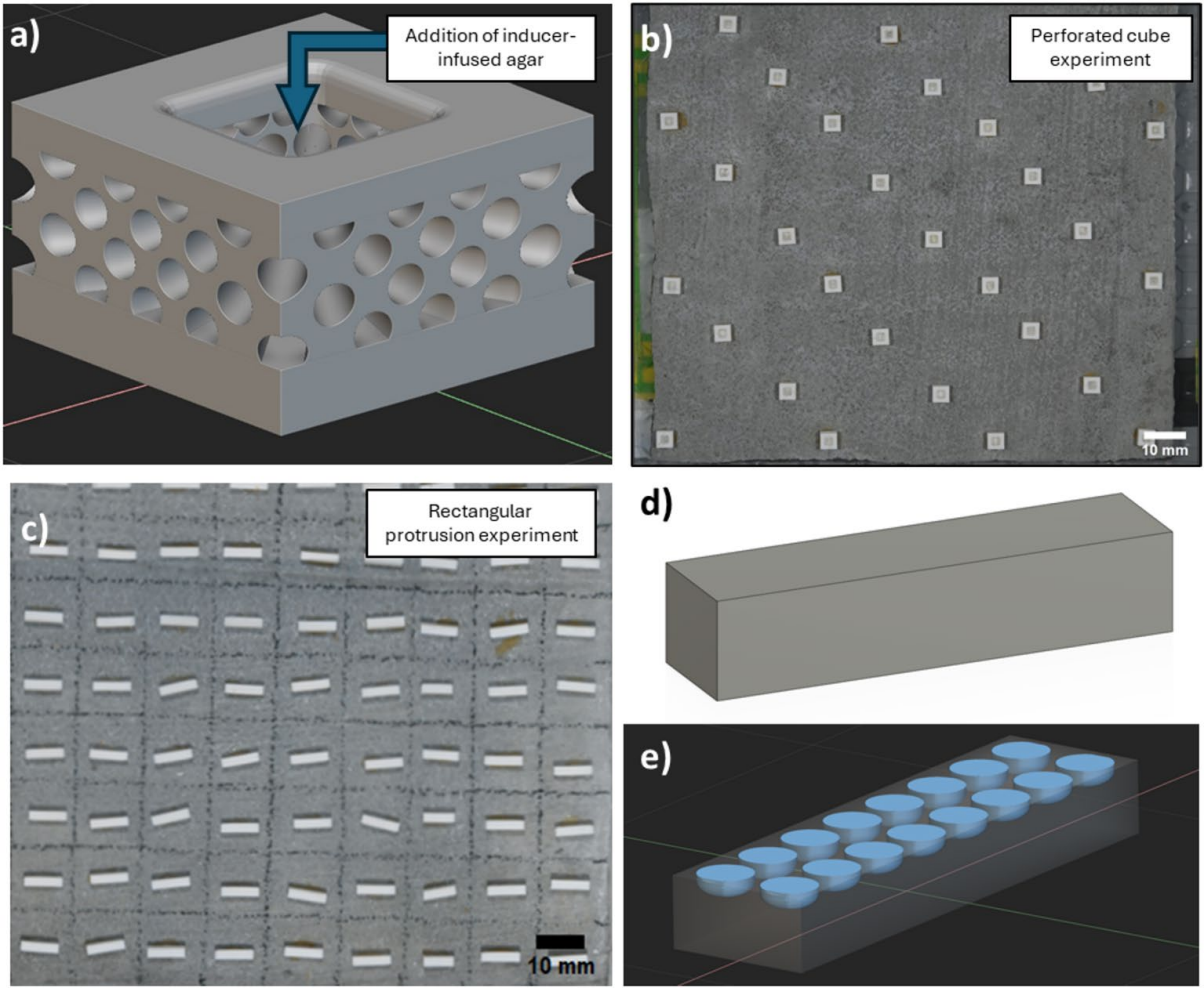
(**a**) Computer-aided design (CAD) representation of alumina ceramic perforated cubes, used to retain chemical inducer in agar for Experiment 2. (**b**) Concrete settlement tile with glued alumina ceramic perforated cubes that contain chemical inducer agar solutions glued onto a concrete tile used in Experiment 2. (**c**) Layout of alumina ceramic protrusions on a portion of concrete tile used in Experiment 3. (**d**, **e**) CAD representations of alumina ceramic protrusions (solid block with no pores, block with 800 µm pores) used in Experiment 3.

The inducer-infused agar was confined within 3D-printed alumina ceramic cubes, which we termed ‘perforated cubes’ due to the presence of cylindrical tubes radiating from the centre of the cube (Fig. 1a). These were then affixed to unconditioned concrete tiles using cyanoacrylate glue (Gorilla Super Glue Gel). Each cube (n = 986) measured 4 × 4 × 3 mm and featured a central well of approximately 13 mm^3^ to hold the inducer-infused agar (Fig. 1a). Alumina ceramic has previously been used in coral settlement successfully, and is valued for its inert properties and durability^25,27^. The cubes radially oriented cylindrical channels extending from the central well (Fig. 1a) were designed using nTopology (V5.4.2). This resulted in highly porous lattice structures with pore diameters ranging from 450 to 600 µm. Steriolithography (SLA) ceramic manufacturing was used to fabricate cubes and protrusions for Experiment 2, and Experiment 3, using a C100 3DCERAM SLA 3D printer. Following printing of the designs for both experiments, the green bodies underwent standard post-processing as outlined by 3DCerams material specification which includes a debinding process undertaken in nitrogen-controlled atmosphere to 600 °C followed by a sintering/densification cycle undertaken in a high temperature standard atmosphere furnace environment (1730 °C for 2 h).

#### Preparation of concrete tiles

The perforated cubes (n = 34) were glued to 14 cm × 14 cm (200 cm^2^) (10 × 10-tabs) unconditioned concrete tiles, with only one treatment per tile, using a minimum amount of cyanoacrylate gel and ensuring an even spatial distribution (Fig. 1b). Glued tiles were allowed to cure for at least 24 h, soaked in fresh water for up to 4 days and rinsed in flowing seawater the day before adding any inducer-infused agar. Treatment agar solutions were freshly prepared on the day of the experiment and heated to 85 °C in a digital dry block heater until use. Approximately 15 µL of each treatment solution was dispensed into the wells and allowed to solidify at room temperature for up to 2 h before being placed into experimental tanks.

#### Experiment 2 treatments

The following treatments were tested in independent tanks: CCA extract (nominal 27% and 53% CCA extract in 1.5% agar) in cubes; Hym-248 (nominal 5 and 10 mM in 3% agar) in cubes; crushed CCA (~ 10 mg per well, suspended in agar) in cubes; unconditioned tile with agar and no inducer in cubes (negative controls); biologically conditioned cubes (positive controls); and live CCA *Porolithon* cf. fragments (positive controls). For CCA extract preparations, 100% extract was dried under a stream of nitrogen, reconstituted with 1.5% agar to achieve desired concentrations, then vortexed and probe-sonicated (Cole-Parmer CP 130, USA, 30% Amplitude) for one minute to ensure homogeneity. Biologically conditioned cubes were included to test the effectiveness of conditioning small, compact objects instead of large, bulky, concrete tiles, and consisted of a tile with previously conditioned cubes (~ 20% mixed-species CCA cover) in one replicate tank, limited by availability of conditioned cubes. Conditioning of these cubes was conducted in a 270 L mesocosm tank dominated by CCA *Lithophyllum* sp. and *Crustaphytum* sp.^25^, after which they were frozen at -20 °C until experimentation^65^. An additional positive control used live *Porolithon* cf. fragments (5 × 5 mm) glued onto unconditioned concrete tiles.

All experimental tiles (n = 34 diagonally spaced perforated cubes or CCA fragments) were placed into their respective treatment tanks, which were 5 L flow-through aquaria (working volume 4.2 L; n_tanks_ = 4 for each treatment, n_tanks_ = 1 for conditioned perforated cubes) with a turnover rate of 0.5 h^-1^, maintained at 27–28 °C and 10–25 µmol photons m^-2^ s^-1^ PAR. Approximately 700 *Acropora kenti* larvae were introduced into each tank with gentle bubbling to prevent surface aggregation of larvae. The total number of settled larvae was counted from images taken 24 h after addition of larvae, which were categorised into four spatial locations: (1) on the cube surface; (2) within the cube well; (3) at the right angle between cube and tile; and (4) on tile at a clear distance from the cube. The total number of spat closely associated with each cube was calculated as the sum of categories 1–3. Settlement success (%) was calculated as the number of settled larvae divided by the total number of larvae added per tank. Parallel well plate assays (10 mL wells) were conducted using the same treatments above (not added to agar), to confirm their capacity to induce larval settlement under controlled conditions.

### Protrusions containing microtopographies with and without biological conditioning (Experiment 3)

#### Preliminary Trials

Preliminary trials were conducted using alumina ceramic or hydroxyapatite rectangular protrusions (2 × 2 × 8 mm) containing circular pores manufactured as described above, hereafter termed ‘protrusions’, each affixed to the surface of unconditioned concrete tiles. Hydroxyapatite was considered for initial investigation due to its natural occurrence as a calcium apatite mineral, its chemical inertness, and environmental compatibility^66^. Pore diameters of 400, 600, 800, and 900 µm, all with a depth of 200 µm, and protrusions without any pores were selected to align with a size range of coral larvae across species^21^. Preliminary trials showed that protrusions with 800 µm diameter pores yielded the highest larval settlement. Thus, Experiment 3 focussed on solid alumina protrusions with or without 800 µm diameter pores.

#### Preparation of concrete tiles

Protrusions (n = 200) were glued to each 28 cm × 14 cm unconditioned concrete tiles (10 × 20-tab layout), with each tile and tank containing only one treatment to prevent cross-contamination of biofilm or CCA cues. Protrusions were affixed using a minimum amount of cyanoacrylate gel onto the centre of every tab (Fig. 1c). Glued tiles were allowed to cure for at least 24 h, soaked in freshwater for up to 4 days and rinsed in flowing seawater the day before adding any larvae to tanks. Biological conditioning of the protrusions occurred over 6 weeks in a 270 L mesocosm tank dominated by CCA *Lithophyllum* sp. and *Crustaphytum* sp.^25^ which yielded 15–30% CCA cover on the protrusions during that time. Conditioned tiles and protrusions were subsequently frozen at -20 °C as per Nordborg et al.^33^.

#### Experiment 3 treatments

Four types of protrusions (n = 2400 total), unconditioned or conditioned and either solid block with no pores or block with 800 µm pores, were glued onto unconditioned tiles to evaluate the effectiveness of microtopographies and localised conditioning in directing settlement of coral larvae. Treatments included: unconditioned tiles with no protrusions (negative controls); biologically conditioned concrete tiles with no protrusions (positive controls); tiles with unconditioned solid block protrusions with no pores (Fig. 1d); tiles with biologically conditioned solid block protrusions with no pores; tiles with unconditioned protrusions with 16 pores (800 µm diameter, 200 µm depth) (Fig. 1e); and tiles with biologically conditioned protrusions with 16 pores (800 µm diameter, 200 µm depth).

Each treatment was replicated in triplicate tanks, which were 15 L flow-through aquaria with a turnover rate of 0.5 h^-1^, maintained at 27–28 °C and 10–25 µmol photons m^-2^ s^-1^ PAR. Immediately after placing settlement tiles within experimental tanks, *A. kenti* larvae (7-d-old, n = 1000) were introduced into each tank. Tiles were removed approximately 40 h post-larval addition and imaged as per Experiment 2. Spat were counted from images and categorised into four spatial locations: (1) on the protrusion surface; (2) within the pore; (3) at the right angle between the protrusion and tile; and (4) on tile at a clear distance from protrusion. The total number of spat associated with each protrusion was calculated as the sum of categories 1–3. Settlement success (%) was calculated as the number of settled larvae divided by the total number of larvae added per tank.

### Image analysis

High-resolution images were taken with a Nikon D810 camera (Nikon AF-S 60 mm f/2.8G ED macro lens with two Ikelite DS161 strobes) mounted on a trolley using CNC software to capture multiple photos at steps along a coordinate grid for exact replication of camera positions. Micro-photographs were captured with a 14MP camera (TOUPCAM L3CMOS) mounted on a Leica MZ6 stereo microscope.

### Data analysis

All statistical analyses and graphical results were performed in RStudio v4.3.2^67^. For Experiment 2, number of spat on each cube was modelled using generalized linear mixed effect models utilising a template model builder (glmmTMB)^68^. Tank was treated as a nested random effect and cubes/CCA fragments as pseudoreplicates within tank (Experiment 2). This method was also used to model spat per tile, with tank as a random effect (Experiment 3). Data were modelled with a negative binomial distribution, inducer treatment as a fixed effect, and cube/CCA fragment nested within tank as a random effect to account for the non-independence of cubes/CCA fragments in the same tank (i.e., same tile). For Experiment 3, settled spat per tile was modelled as above using a negative binomial distribution with tile treatment as a fixed effect and tank as a random effect. Data were modelled in Experiment 3 at the level of spat per tile to compare to positive and negative controls, which had no protrusions or CCA fragments affixed. A null model was formulated using only random effects for both Experiment 2 and Experiment 3 to compare with the generalized linear mixed effect models. Model assumptions were assessed and validated using DHARMa residual analysis^69^, results were visualised using ‘ggplot2’^70^, and model selection was undertaken by comparing models with different distributions against the null model, using second-order Akaike Information Criterion (AICc) in the MuMIn package^71^.

## Results and discussion

### Efficacy of chemical inducers (Experiment 1)

Settlement was not observed in negative control well plate assays, apart from *Fungia fungites* larvae. A mean settlement success of 14% ± 10 SE of *F. fungites* larvae was observed in the absence of a cue; therefore, that assay was removed from further data analysis and consideration. Notably, mean settlement in *F. fungites* with 1 mM Hym-38 was double (29% ± 5.2) that of settlement in the negative controls.

A summary of settlement success across species and active treatments is presented in Table 1, with the responses to all treatment concentrations detailed in Supplementary Table 3. Among the six neuropeptides tested, Hym-248 was the most active, inducing settlement behaviour in all replicates for 7 of the 13 species that required settlement cues. Across the two concentrations tested, Hym-248 induced a mean settlement success of 68% ± 5.6 SE, which was intermediate between the mean settlement rates for CCA (74% ± 6.7) and rubble fragments (57% ± 8.2) (Table 1). Hym-38 was the only other active neuropeptide, inducing settlement in four acroporids, including greater than 40% settlement in *A. millepora* and *A. spathulata* (Table 1). Of the seven members of the Acroporidae family tested, only *A. loripes* and *M. turtlensis* did not respond to Hym-38 (Table 1, Supplementary Table 3). There were some sporadic, low settlement responses of some acroporids to Hym-53, Hym-54 and Hym-249 (Supplementary Table 3). The only non-acroporid to show a response to these peptides was *Dipsastrea speciosa*, which showed irregular settlement (≤ 10%) with an average (± SE) of 8.3% ± 6.8 to Hym-248.

**Table 1:** Mean settlement success (%) ± SE of CCA, rubble fragment, and two GLWamide neuropeptides (at two concentrations) in coral larvae. Blank cells represent no observed larval settlement. Settlement success using all chemical inducers and results for *F. fungites* are listed in Supplementary Table 3.

Treatment							
Species	Family	CCA	Rubble	Hym-38 1mM	Hym-38 10mM	Hym-248 1mM	Hym-248 10mM
*Acropora austera*	Acroporidae	44.8 ± 13.3	53.3 ± 1.4		22.5 ± 11.2	33.3 ± 2.5	18.1 ± 6.2
*Acropora kenti*	Acroporidae	90 ± 0	13.3 ± 10.9		14.8 ± 12.1	60 ± 0	53.3 ± 3.5
*Acropora loripes*	Acroporidae	15.7 ± 6.5	23.8 ± 10.3			55.3 ± 9.1	48.5 ± 6
*Acropora* aff.* kenti*	Acroporidae	97 ± 2.4	55 ± 3.5			93.3 ± 5.4	46.0 ± 16.1
*Acropora millepora (*October collection*)*	Acroporidae	93.3 ± 2.7	93.3 ± 5.4	43.7 ± 13.3	51.1 ± 11.9	68.9 ± 6.8	58.9 ± 4
*A. millepora (*November collection*)*	Acroporidae	83.8 ± 9.1	23.1 ± 10.5	3.3 ± 2.7	40.0 ± 8.2	100 ± 0	58.1 ± 6.1
*Acropora spathulata*	Acroporidae	84.2 ± 3.8	13.3 ± 7.2	38.5 ± 15.8	41.5 ± 1.2	85.5 ± 3.2	50 ± 4.7
*Dipsastrea speciosa*	Merulinidae	72.7 ± 15.5	83.6 ± 9.9		8.3 ± 6.8		
*Echinophyllia orpheensis*	Lobophyllidae	86.7 ± 5.4	86.1 ± 6				
*Goniastrea retiformis*	Merulinidae	100 ± 0	100 ± 0				
*Lobophyllia corymbosa*	Lobophyllidae	89.3 ± 5.2	28.7 ± 9.3				
*Montipora turtlensis*	Acroporidae	19 ± 10.8	27.8 ± 6				22.4 ± 5.1
*Mycedium elephantotus*	Merulinidae	82.5 ± 2	96.7 ± 2.7				
*Platygyra daedalea*	Merulinidae	92.7 ± 3.3	87.3 ± 7.3				

The neuropeptide Hym-248 and other GLWamide peptides are thought to act as endogenous communication molecules, transmitting internal signals that trigger metamorphosis in Cnidaria, including hydrozoans and corals, following detection of exogenous settlement cues^47,61,72^. In our experiments, Hym-248 likely bypasses the need for a natural cue, directly triggering the larvae’s metamorphic program^47^. At the molecular level, GLWamide peptides are hypothesised to act via G-protein-coupled neuropeptide receptors, activating downstream sensory and developmental signalling pathways that include neuronal circuits and conserved morphogenetic regulators such as Wnt/Frizzled signalling^61,72,73^. Transcriptomic analyses of Hym-248-induced metamorphosis in *Acropora* further implicate GPCR-mediated sensory pathways, including putative neurotransmitter (e.g. GABAergic) and developmental receptors, in initiating the irreversible transition from larva to primary polyp^73^. Previous studies have shown that Hym-248 was the only GLWamide neuropeptide that systematically triggered a response from acroporid corals, although not in all genera of the family^46,47^. Settlement success, although low (≤ 10%), has been observed in several non-acroporid species, including *Diploastrea heliopora* (Diploastraeidae), *Galaxea fascicularis* (Euphylliidae), *Oulophyllia crispa* (Lobophyllidae*),* and *Porites cylindrica* (Poritidae)^21^. However, in this study Hym-248 did not induce a settlement response in tested species from the families Merulinidae or Lobophyllidae, suggesting that this specific metamorphic pathway may not be consistently retained in non-acroporids. Nevertheless, findings from this and previous studies^47,46,21^ support the potential application of this neuropeptide to induce acroporid larval settlement on seeding surfaces for coral restoration.

Dopamine has previously been reported to induce settlement in *Leptastrea purpurea*^45^. However, none of the 13 species tested here responded to dopamine at comparable exposure concentrations (Supplementary Table 3), suggesting it is unlikely to be a suitable candidate for restoration purposes. Further testing across a broader concentration range and additional coral families may still be warranted. Notably, water in dopamine assays showed visible discolouration after 24 h, consistent with oxidation to dark transformation products previously observed under light at pH > 7^74^, which may have impacted larval settlement. Only *A. spathulata* larvae responded to epinephrine, which has also been shown to induce settlement in *L. purpurea*^45^. However, settlement success remained low, averaging 3.3% ± 2.1 SE and 2.6% ± 2.7 at 1 and 10 mM, respectively (Supplementary Table 3).

By contrast, CCA and rubble fragments induced substantially higher settlement, averaging 74% ± 6.7 SE and 57% ± 8.2, respectively, across all coral species excluding *Fungia fungites*. Settlement remained relatively low (< 70%) in these positive controls for *Montipora turtlensis*, *Acropora austera* and *A. loripes* (Table 1)*.* Potential explanations include mismatched settlement cues among species, suboptimal larval condition, or premature use of larvae. For example, Abdul Wahab et al.^19^ found higher larval settlement with the CCA *Lithophyllum* than *Porolithon* cf. *onkodes* with *Montipora aequituberculata,* potentially explaining the low larval settlement in *M. turtlensis* in this study. Whitman et al.^22^ reported that *Porolithon onkodes* induced > 50% settlement in *A. austera* and *A. loripes;* however, both *A. austera* and *A. loripes* are known to have long pre-competency periods (> 5 d)^21^, so some 5- and 6-d-old larvae may not have been fully competent to settle despite positive results in pre-experiment competency assays conducted the day prior.

### Embedded chemical inducers (Experiment 2)

Larval settlement in response to embedded chemical inducers was concentration-dependent, with greater settlement success as a response to the higher concentration of Hym-248 and CCA extract (Table 2). Both inducers achieved mean settlement of ~ 24%, about half the level observed in the positive control (Table 2). Tiles with biologically conditioned cubes and live CCA fragments induced the highest settlement (46.5%, Table 2). All treatments except crushed CCA significantly promoted settlement relative to the negative control (Supplementary Figure S1). Except for biologically conditioned cubes, all treatments also differed significantly from the CCA fragment positive control (*p* < 0.05). Pairwise comparisons confirmed that CCA extract, Hym-248, and biologically conditioned cubes effectively induced settlement (*p* < 0.05), while crushed CCA did not (Supplementary Figure S2). No significant difference was observed between live CCA fragments and biologically conditioned cubes (*p* > 0.05), indicating that the latter achieved settlement rates similar to the positive control (Supplementary Figure S2). In stark contrast to parallel well plate settlement assays where crushed CCA induced approximately 75% settlement (Supplementary Figure S1), very few larvae settled in response to crushed CCA retained in agar within cubes (Table 2). This reduced efficacy is likely due to retention of biochemical cues within the agar matrix, limiting their bioavailability to larvae. As expected, settlement was negligible (0.04%) in the absence of chemical or physical cues (Table 2).

**Table 2:** Mean (± SE) settlement success (in % of total larvae) of *Acropora kenti* larvae associated with alumina ceramic perforated cubes with varying chemical inducers constrained within porous wells and CCA fragments (positive controls). Measures include average spat per perforated cube and the percent of spat associated with cubes relative to the total settled spat. n denotes number of tiles (10 × 10 tabs) per treatment.

Treatment	% Settlement success (mean ± SE)	Spat per cube (mean ± SE)	Spat associated with a cube relative to total settlers (%)
Negative control (no inducer) (n = 4)	0.036 ± 0.003	0 ± 0.01	100
Positive control (CCA fragments) (n = 4)	46.4 ± 0.5	9.6 ± 1.2	98.7
CCA extract—27% (n = 4)	16.9 ± 0.4	3.5 ± 1	74.6
CCA extract—53% (n = 4)	23.9 ± 1	4.9 ± 2.5	93.6
Crushed CCA (n = 4)	0.29 ± 0.01	0 ± 0.03	25.0
Hym-248—5 mM (n = 4)	10.0 ± 0.2	2.1 ± 0.5	54.8
Hym-248—10 mM (n = 4)	23.8 ± 0.2	4.9 ± 0.0.6	39.1
Biologically conditioned cubes (n = 1)	46.7	9.6	98.8

**Fig. 2:**
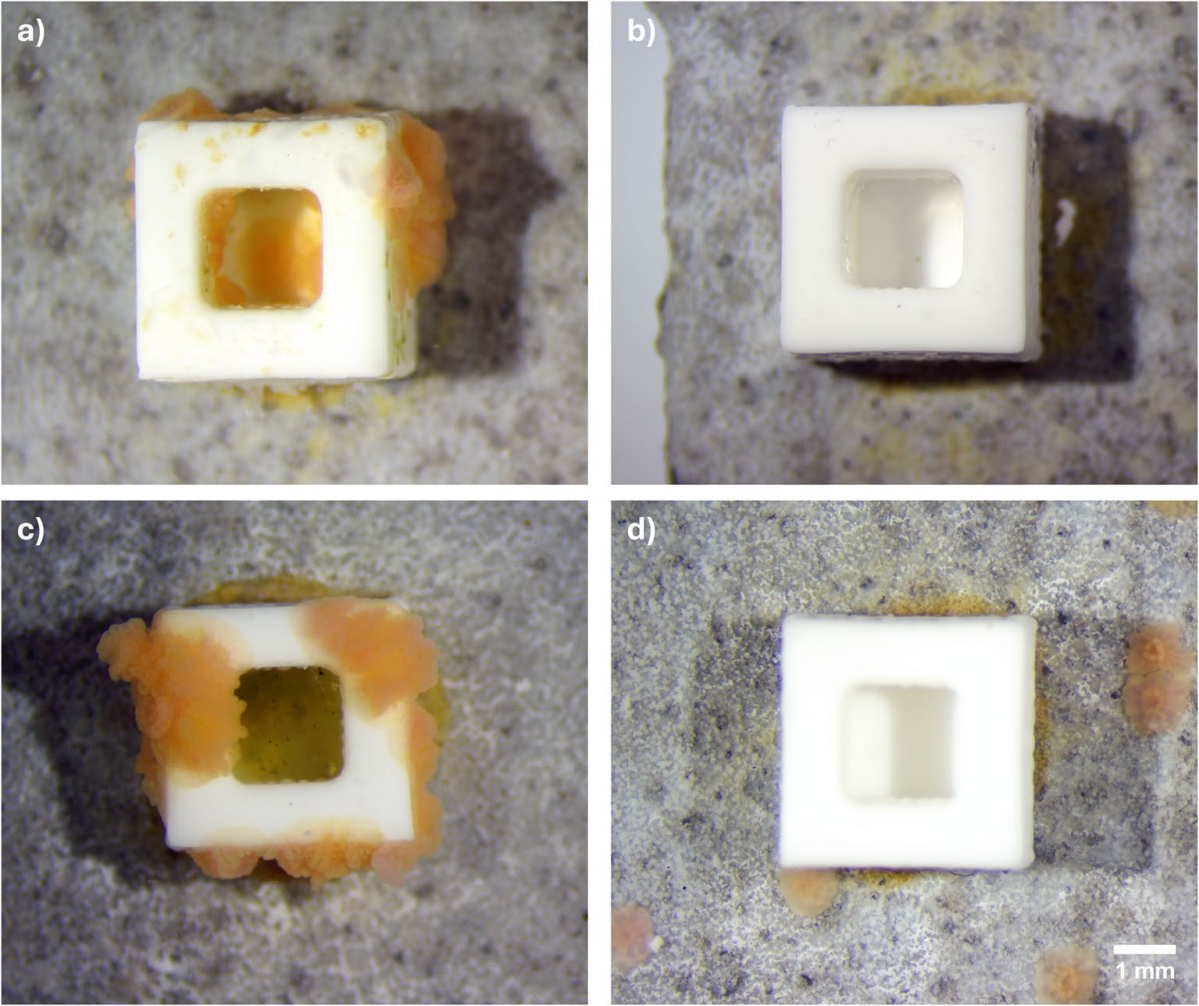
Experiment 2: *Acropora kenti* larvae were introduced to 3D-printed alumina ceramic perforated cubes (4 × 4 × 3 mm) to retain chemical inducers in agar. (**a**) biologically conditioned cube with the addition of agar but no chemical inducer within agar; (**b**) unconditioned cube with the addition of agar but no chemical inducer within agar; (**c**) unconditioned cube with the addition of agar containing CCA extract (53%); (**d**) unconditioned cube with the addition of agar containing neuropeptide Hym-248 (10 mM). Each tank contained a tile with a single cue treatment. Scale bar = 1 mm.

The vast majority of spat (> 98%) settled immediately adjacent to or on conditioned cubes and CCA fragments (Table 2, Fig. 2a), consistent with patterns previously observed in well-plate assays using CCA fragments^35^. Negligible settlement (< 1%) was observed in or adjacent to cubes lacking a chemical cue (Table 2, Fig. 2b). High proportions of spat (75–95%) also settled on or adjacent to cubes containing CCA extract-infused agar, often forming dense aggregations (Table 2, Fig. 2c). This gregarious settlement behaviour with conspecifics, documented in several coral species^75–77^. In contrast, larvae exposed to Hym-248-infused agar exhibited more solitary settlement, with only 39–55% of spat located near cubes, and a more even distribution across tile surfaces (Table 2, Fig. 2d). Differences in solitary versus gregarious settlement behaviour may have important consequences for genetic structure and the formation of chimeric colonies in larval-based restoration^78^. Gregarious settlement increases the likelihood of conspecific aggregation and chimera formation, which has been associated with enhanced survivorship^78–82^ but also reduced individual polyp size in some coral species^77^. This suggests a potential trade-off between increased areal footprint and early survivorship in aggregated settlers versus larger polyp size in solitary recruits, with currently unresolved implications for long-term fitness, immunity, stress tolerance, and growth. The preferential promotion of solitary or clustered settlement is likely to be context-dependent, varying with environmental conditions, post-settlement competition, and restoration objectives^76^. While the present study demonstrates that settlement behaviour can be influenced by the type of chemical cue provided, experimentally evaluating the downstream ecological and genetic consequences of inducible settlement modes represents an important avenue for future research.

Overall, higher concentrations of CCA extract and Hym-248 increased settlement, and settlement consistently occurred adjacent to the source of the inducer, indicating successful immobilisation within the agar matrix. Larvae likely responded either to direct contact with the embedded cues or to locally elevated concentrations diffusing from the matrix. These results demonstrate that both inducers retained biological activity despite the heating required to dissolve the agar. Retention in agar is essential for water-soluble inducers, which would otherwise rapidly dissolve and become diluted under the high-volume, flow-through conditions required for large-scale spat settlement. Spatially constraining the inducer within the porous cavities of 3D-printed ceramic protrusions improved local retention and target settlement outcomes.

Directing settlement onto large unconditioned concrete tiles offers a promising strategy to reduce the logistical and resource demands of large-scale restoration. Early efforts to immobilise chemical inducers included a ‘chemical flypaper’ approach, which bound CCA cell-wall polysaccharides, a different class of chemical inducers, to hydrophobic-interaction chromatography resin^17^. This method successfully induced settlement of *Agaricia humilis* larvae on artificial substrates, but extraction and purification was considerably more complex and yielded inducer fractions with lower activity compared to the simpler alcoholic extraction of CCA^35^ used in the present study. More recently,^51^ immobilised semi-purified exometabolites from the CCA *Hydrolithon reinboldii* onto non-porous silica nanoparticles embedded in a hydrogel matrix, creating a delivery system known as ‘SNAP-X’. This was used to settle *Montipora capitata* larvae in both static well-plate assays and small-scale (0.5 L) flow-through systems. Together, these advances highlight the potential for conditioning-free settlement strategies, but also the need for continued optimisation of immobilisation methods to improve inducer retention, bioavailability, and delivery efficiency at scale. Importantly, such methods can be extended to other classes of chemical inducers as they are identified, broadening the toolkit for targeted larval settlement in coral restoration.

### Protrusions with microtopographies (Experiment 3)

**Fig. 3:**
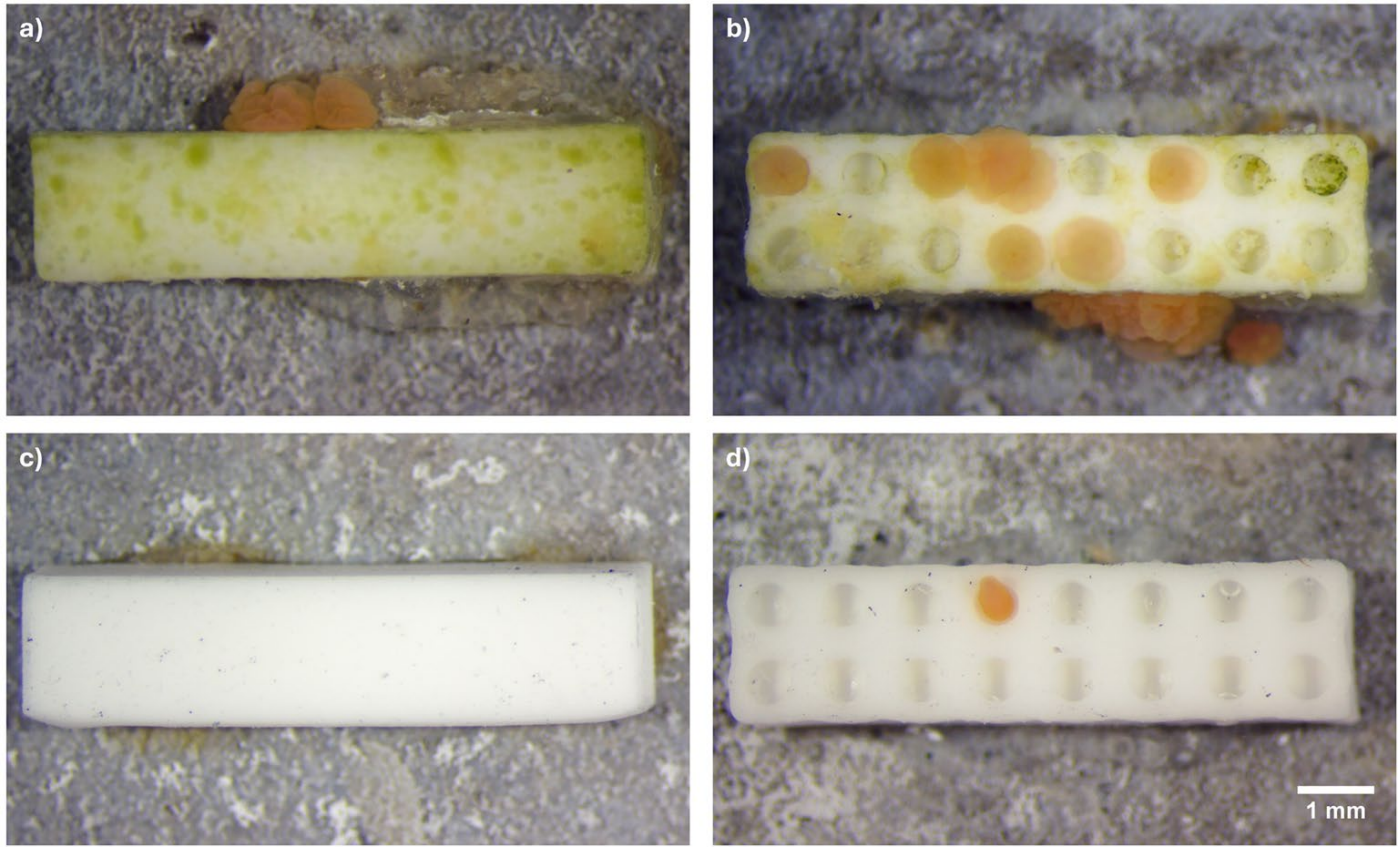
*Acropora kenti* larvae were introduced to 3D-printed alumina ceramic rectangular protrusions (2 × 2 × 8 mm). (**a**) 6-week biologically conditioned solid protrusion with no pores; (**b**) 6-week biologically conditioned protrusion with 800 µm pores. (**c**) unconditioned solid protrusion with no pores; (**d**) unconditioned protrusion with 800 µm pores. Note that *A. kenti* larvae settled within pores in (**b**) and were exploring pores in (**d**) i.e., not attached.

Conditioned protrusions on unconditioned concrete tiles induced approximately 50% larval settlement (≈ 2.5 spat per protrusion, Table 3, Fig. 3a,b), which exceeded settlement on the positive control (41%). In contrast, settlement was negligible on unconditioned tiles (< 0.1%) and unconditioned protrusions (< 1%) (Table 3, Fig. 3c,d). Settlement success on unconditioned tiles with protrusions was significantly different than on live tiles (*p* < 0.05), whereas biologically conditioned protrusions were not significantly different (*p* > 0.05). Pairwise comparisons confirmed no significant effects of pores alone, either on unconditioned or conditioned protrusions (*p* > 0.05).

**Table 3:** Mean (± SE) settlement success (in % of total larvae) of *Acropora kenti* associated with biologically conditioned or unconditioned alumina ceramic protrusions. Other measures include the number of spat per protrusion, the percent of *A. kenti* spat associated with protrusions relative to the total spat, percent settlement within a microtopographic pore, and percent of tabs that had at least one spat (mean ± SE). n denotes number of tiles (10 × 20 tabs) per treatment. NA—Not applicable.

Treatment	Pore size (µm)	% Settlement success (mean ± SE)	Spat per protrusion (mean ± SE)	Spat associated with protrusion (%)	Settlement in pore (%)	Tabs with at least one spat (% mean ± SE)
Negative control (n = 3)	NA	0.067 ± 0.07	NA	NA	NA	NA
Positive control (n = 3)	NA	41 ± 5.5	NA	NA	NA	NA
Conditioned (n = 3)	0	50.9 ± 10.4	2.5 ± 0.4	99.1	NA	50.5 ± 1.5
Conditioned (n = 3)	800	47.1 ± 5.2	2.4 ± 0.2	100	24.7	52.2 ± 3.2
Unconditioned (n = 3)	0	0.27 ± 0.2	0 ± 0.01	88	NA	1.2 ± 0.9
Unconditioned (n = 3)	800	0.90 ± 0.3	0 ± 0.01	94.4	59.3	3.2 ± 0.6

Biological conditioning was the primary driver of settlement. Nearly all spat (99%) settled directly on or adjacent to conditioned protrusions, with ~ 25% of these within pores (Table 3, Fig. 3b). Although unconditioned protrusions induced only limited settlement (27 settlers in total), ~ 60% settled within pores (Fig. 2d), suggesting pore microstructure may provide a subtle physical cue in the absence of biological inducers. While previous studies have reported settlement induced solely by microscale surface features^57,83^, this effect appears minimal for *A. kenti*. Nevertheless, coral larvae are known to settle in crevices and interstitial reef spaces^13^, consistent with the ‘Attachment Point Theory’^84^ that suggests larvae preferentially settle in topographic features matching their size^57^. Given *A. kenti* larvae average ~ 500 ± 70 µm in diameter^85^, the 800 µm diameter, 200 µm deep pores likely offer appropriately scaled refuges.

Approximately half of the conditioned rectangular protrusions (200 per tile) supported at least one spat, with settlement strongly concentrated at the centre of each 14 × 14 mm tab (Table 3). Since these tabs are designed to be broken off for insertion into ceramic deployment devices, settlement on edges increases the risk of spat lost during handling. Centralised settlement, therefore, enhances system efficiency by retaining viable spat, and directing larvae to mm scale topography, or optimal settlement locations. Reducing larval density may further increase the proportion of tabs with settlers by limiting gregarious settlement. Additional optimisation is required to promote more uniform settlement and reduce wasted substrate.

## Conclusion

This study expands the taxonomic range of coral responsive to Hym-248 and Hym-38 and demonstrates, for the first time, that larval settlement can be reliably and spatially directed by immobilising known chemical cues and conditioning discrete microtopographic features on 3D-printed substrates. By decoupling settlement control from whole-substrate biological conditioning, this approach introduces a new level of precision and flexibility into coral aquaculture workflows, with clear implications for improving productivity at scale.

A key outcome of this work is the demonstration that effective settlement can be achieved without conditioning entire settlement tiles or maintaining large conditioning systems. Instead, settlement can be directed to small, engineered features, allowing conditioning effort to be concentrated on discrete protrusions rather than large substrate surfaces. Compared to traditional concrete tiles, biologically conditioning small protrusions such as those used in this study requires substantially less aquarium space: 64 cm^2^ of tank area yielded 200 conditioned protrusions, whereas approximately 580 cm^2^ is required to condition 200 conventional concrete tabs, representing an approximate nine-fold reduction in conditioning footprint.

Beyond space efficiency, directing larvae to predefined locations improves operational efficiency throughout the aquaculture pipeline. Predictable, centralised settlement reduces loss of spat during handling and tab separation, increases the proportion of usable settlement units, and simplifies integration with existing deployment devices. Collectively, these factors reduce human intervention per recruit produced and improve consistency across production batches, key constraints in large-scale restoration programs. The extent to which these efficiency gains can be realised will depend on a range of factors, including coral species, settlement behaviour, inducer efficacy, and the configuration and capacity of existing and future aquaculture infrastructure. Importantly, this study addresses one component of the production pipeline, larval settlement, and does not resolve downstream processes that ultimately contribute to restoration success. Post-settlement survival and growth are influenced by additional interacting factors, including fouling pressure, grazing, hydrodynamic conditions, and husbandry practices, which were not evaluated here and warrant dedicated, longer-term investigation.

Overall, this study establishes a framework for directing coral larval settlement using engineered substrates that substantially reduce aquarium space, conditioning effort, and handling demands. By improving the efficiency with which settlement substrates are prepared, used, and integrated into deployment workflows, these methods provide a practical pathway to increasing the scalability and cost-effectiveness of sexually propagated coral aquaculture for reef restoration.

## Supplementary Information

Supplementary Information.

## Data Availability

All data are freely available and housed in the AIMS data repository: https://tsv-apps.aims.gov.au/metadata/view/383f7436-645b-43b0-9721-dc9172f32463.
