# Supplementary material for: Directing coral larval settlement in coral aquaculture for reef restoration
Source: Sci Rep. 2026 Feb 5;16:7358. doi: 10.1038/s41598-026-37592-x (PMC12923894; doi:10.1038/s41598-026-37592-x)
Supplement: Supplementary file 1 — Supplementary Information. [file 41598_2026_37592_MOESM1_ESM.pdf]

Supplementary Information

Directing coral larval settlement in coral aquaculture for reef restoration

Nico D. Briggs[1], Andrew P. Negri[1,2], Elsa Antunes[3], Matthew Drane[3], Andrea Severati[1], *Florita Flores[1,2]

[1]Australian Institute of Marine Science, Townsville QLD 4810, Australia
[2]AIMS@JCU, Division of Research & Innovation, James Cook University and Australian Institute of Marine Science, Townsville QLD 4810, Australia
[3]College of Science and Engineering, James Cook University, Townsville QLD 4811, Australia

Correspondence:

PMB No. 3, Townsville MC, Townsville QLD 4810, Australia

*f.flores@aims.gov.au

Supplementary Table S1. Coral species and spawning information used in well plate settlement assays to compare chemical inducers (Experiment 1). A. kenti *collected in November 2024 were used for Experiments 2 and 3. DAFM = days after full moon.*

Family	Species	Spawning date	DAFM	Larval culture tank size	No. colonies per culture	Larval age used in tests	Collection location	Collection permits	Reproductive Method
Acroporidae	*Acropora austera*	30/11/23	2	70L	5	6	Davies Reef	G21/38062.1	Hermaphroditic
Acroporidae	*Acropora kenti*	2/12/23	10	70L	5	5	Falcon/Esk Reefs	G23/ 49457.1	Hermaphroditic
Acroporidae	*Acropora kenti*	21/11/24	5	500L	7	7	John Brewer Reef	G23/ 49457.1	Hermaphroditic
Acroporidae	*Acropora loripes*	6/12/23	8	500L	6	5	Davies Reef	G21/45348.1	Hermaphroditic
Acroporidae	*Acropora millepora*	31/10/23	2	500L	11	4	Falcon Reef	G23/ 49085.1	Hermaphroditic
Acroporidae	*Acropora millepora*	30/11/23	2	70L	7	6	Falcon Reef	G23/ 49457.1	Hermaphroditic
Acroporidae	*A.* aff.*kenti*	2/11/23	4	70L	4	5	Falcon Reef	G21/45348.1	Hermaphroditic
Acroporidae	*Acropora spathulata*	30/11/23	2	500L	12	7	Davies Reef	G21/ 45348.1, G23/ 49457.1	Hermaphroditic
Merulinidae	*Dipsastraea speciosa*	3/12/23	5	70L	8	4	Davies Reef	G21/45348.1, G23/ 49457.1	Hermaphroditic
Lobophylliidae	*Echinophyllia orpheensis*	3/12/23	5	70L	5	5	Esk Reefs	G23/ 49457.1	Hermaphroditic
Merulinidae	*Goniastrea retiformis*	2/12/23	4	70L	12	4	Falcon/Esk Reefs	G21/38062.1	Hermaphroditic
Lobophylliidae	*Lobophyllia corymbosa*	3/12/23	5	70L	8	5	Esk Reefs	G23/ 49457.1	Gonochoric
Acroporidae	*Montipora turtlensis*	9/12/23	11	70L	3-15	5	Davies Reef	G23/ 49457.1	Hermaphroditic
Merulinidae	*Mycedium elephantotus*	3/12/23	5	70L	4	4	Seasim	None - Seasim Stock	Hermaphroditic
Merulinidae	*Platygyra daedalea*	2/11/23	4	70L	6	6	Falcon/Esk Reefs	G21/45348.1, G23/ 49085.1	Hermaphroditic

Supplementary Table S2. Structure, sequence and purity of GLWamide-family neuropeptides used in well plate settlement assays in Experiment 1.

ID	Primary structure	Sequence	Purity (%)	Supplier
Hym-38	GPPhPGLW-NH2	Gly-Pro-Pro-Pro-Gly-Leu-Trp	98.5	NovoPro Bioscience Inc
Hym-53	NPYPGLW-NH2	H-Glu-Pro-Leu-Pro-Ile-Gly-Leu-Trp-[illegible]	98	NovoPro Bioscience Inc
Hym-54	GPMTGLW-NH2	H-Gly-Pro-Met-Thr-Gly-Leu-Trp-NH2	98.5	NovoPro Bioscience Inc
Hym-248	EPLPIGLW-NH2	H-Asn-Pro-Tyr-Pro-Gly-Leu-Trp-NH2	> 95	Sigma Genosys
Hym-249	KPIPGLW-NH2	H-Lys-Pro-Ile-Pro-Gly-Leu-Trp-OH	99	NovoPro Bioscience Inc
Hym-331	GPPPGLW-NH2	Gly-Pro-Pro-Pro-Gly-Leu-Trp-NH2	98.4	NovoPro Bioscience Inc

*Supplementary Table S3. Mean settlement success (% ± SE) of CCA, rubble fragment, dopamine, epinephrine and six GLWamide neuropeptides on coral larvae. *denotes corals collected from Palm Island Group while no star denotes corals collected from Davies Reef. **denotes corals maintained at the AIMS National Sea Simulator. Blank cells represent no observed larval settlement.*

**Treatment**	**CCA**	**Rubble**	**Dopamine 1mM**	**Dopamine 10mM**	**Epinephrine 1mM**	**Epinephrine 10mM**	**Hym-38 1mM**	**Hym-38 10mM**	**Hym-53 1mM**	**Hym-53 10mM**	**Hym-54 1mM**	**Hym-54 10mM**	**Hym-248 1mM**	**Hym-248 10mM**	**Hym-249 1mM**	**Hym-249 10mM**	**Hym-331 1mM**	**Hym-331 10mM**
Acropora austera	44.8 ±13.3	53.4 ±1.4						22.5 ±11.2		6.7 ±2.5			33.3 ±2.5	18.2 ±6.2				
*Acropora kenti**	90 ±0	13.3 ±10.9						14.8 ±12.1					60 ±0	53.3 ±3.5				
Acropora loripes	25 ±6.5	23.8 ±10.3											55.3 ±9.1	48.5 ±6				
Acropora millepora Oct collection*	93.3 ±2.7	93.3 ±5.4					43.7 ±13.3	51.1 ±11.9	3.7 ±3		7.4 ±1.7		68.9 ±6.9	58.9 ±4				
Acropora millepora Nov collection*	93.8 ±9.1	23.2 ±10.5					3.3 ±2.7	40 ±8.2					100 ±0	58.2 ±6.1		15.2 ±12.4		
A. aff. *kenti*	97 ±2.5	55 ±3.5							23.3 ±19.1				93.3 ±5.4	46 ±16.1				
Acropora spathulata	84.2 ±3.8	13.3 ±7.2			2.6 ±2.7	3.3 ±2.1	38.5 ±15.8	41.5 ±1.2					85.6 ±3.2	50 ±4.7				
Dipsastrea speciosa	72.7 ±15.5	83.6 ±9.9						8.3 ±6.8										
*Echinophyllia orpheensis**	86.7 ±5.4	86.1 ±6																
Fungia fungites	60.9 ±11.3	67.5 ±9.6	7.1 ±3.4	9.4 ±5.2	12.1 ±1.5	2.4 ±1.9	29.4 ±5.1	10.1 ±2.7	7.5 ±3.6	5.3 ±2.2	19.2 ±3	14.6 ±5	15.9 ±4.3	16 ±4.8	9.8 ±1.9	9.2 ±4.8	13.9 ±4	7.1 ±7.6
*Goniastrea retiformis**	100 ±0	100 ±0																
*Lobophyllia corymbosa**	89.3 ±5.3	28.7 ±9.3																
Montipora turtlensis	19 ±10.1	27.8 ±6												22.4 ±5.1				
*Mycedium elephantotus***	82.5 ±2	96.7 ±2.7																
*Platygyra daedalea**	92.7 ±3.3	87.3 ±7.3																

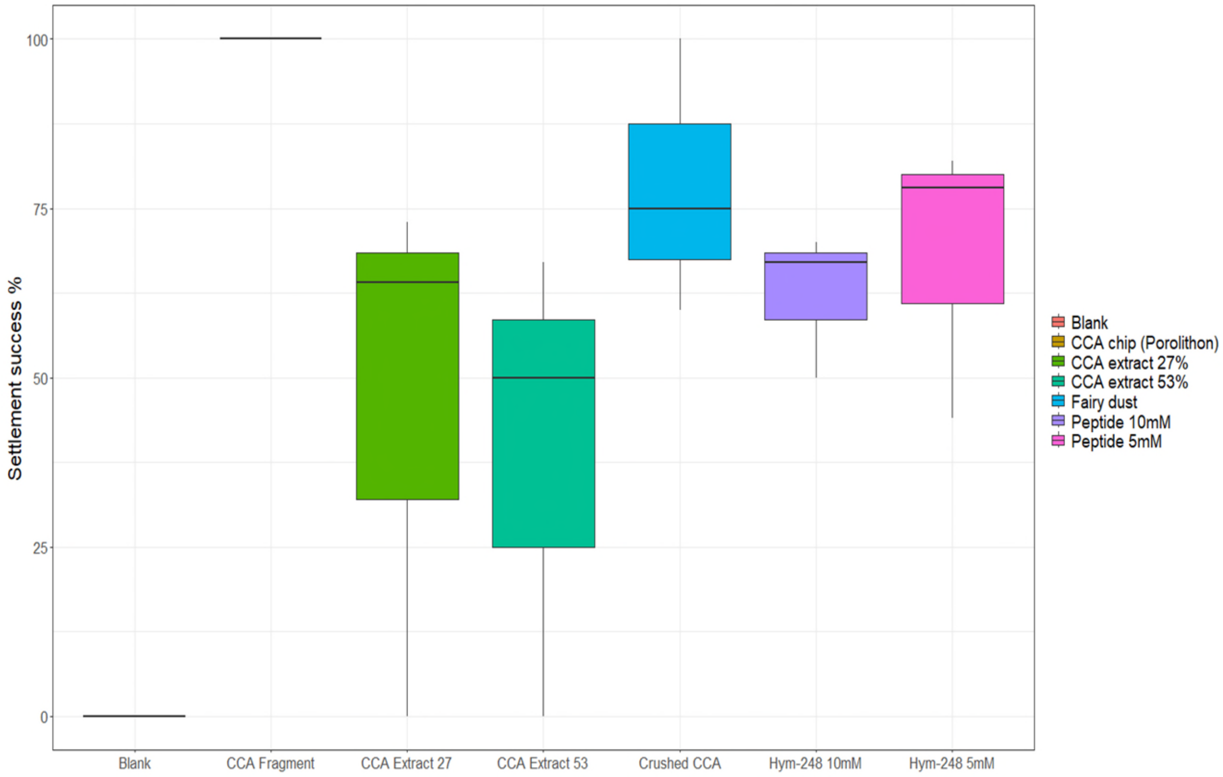

Supplementary Figure S1. Boxplot showing settlement response (%) of Acropora kenti *to chemical inducers conducted in 6-well plates performed in parallel with flow-through Experiment 2 (with protrusions).*

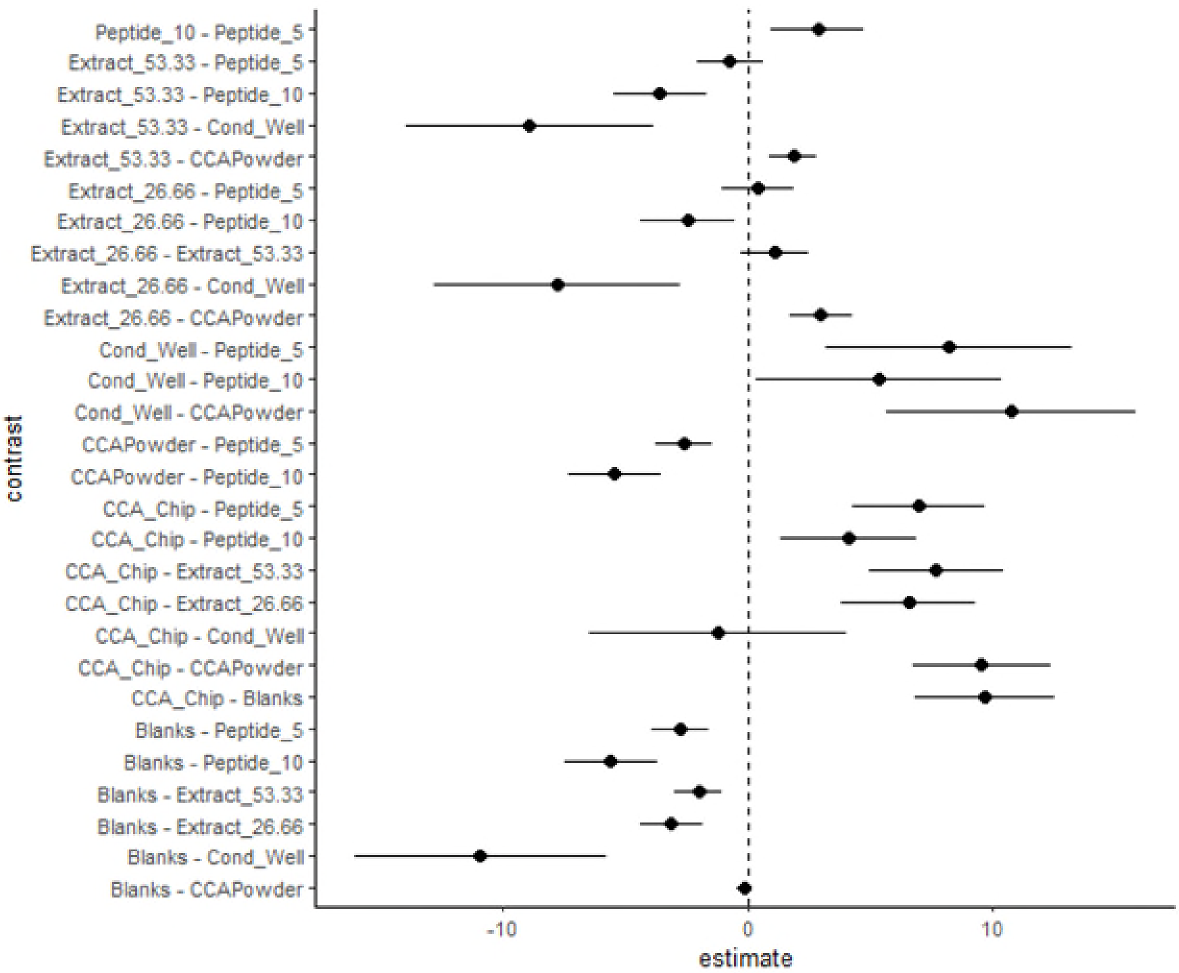

Supplementary Figure S2. Pairwise contrasts of treatments (Experiment 2). The dot on each line is the point estimate of the difference in mean spat per well and horizontal lines extending from the point represent 95% upper and lower confidence intervals.
